# Improving Clinicians’ Implementation of Guidelines to Help Women Stop Smoking in Pregnancy: Developing Evidence-Based Print and Video Materials

**DOI:** 10.3390/ijerph181910522

**Published:** 2021-10-07

**Authors:** Jo M. Longman, Catherine Adams, Christine Paul, James McLennan, Megan E. Passey

**Affiliations:** 1 University Centre for Rural Health, The University of Sydney, P.O. Box 3074, Lismore, NSW 2480, Australia; megan.passey@sydney.edu.au; 2 Northern New South Wales Local Health District, Locked Mail Bag 11, Lismore, NSW 2480, Australia; Catherine.Adams2@health.nsw.gov.au; 3 School of Medicine and Public Health, University of Newcastle, Callaghan, NSW 2308, Australia; chris.paul@newcastle.edu.au; 4 Alcohol and Drug Service, St Vincent’s Health Network, 390 Victoria Street, Darlinghurst, NSW 2010, Australia; james.mclennan@svha.org.au

**Keywords:** smoking in pregnancy, smoking cessation, antenatal care, Behaviour Change Wheel, Theoretical Domains Framework, materials development, stakeholder engagement

## Abstract

Smoking in pregnancy remains a public health challenge. Our team developed a comprehensive intervention using the Behaviour Change Wheel to support clinicians’ implementation of guidelines on supporting women to stop smoking in pregnancy. Integral to the intervention was a suite of evidence-based video and print materials. This paper describes the rationale and process for developing these materials. Comprehensive mixed methods research was undertaken to identify the key barriers and enablers for clinicians in implementing the guidelines. This research identified which behaviours required change, and which behaviour change techniques were best suited to effecting that change. Materials were developed based on this understanding, in a collaborative process with multiple stakeholders, and their feasibility and acceptability explored in a small trial. Materials developed included leadership, clinician and client resources. There are considerable advantages to systematically and collaboratively developing materials which are integral to a behaviour-change intervention even though it is resource intensive to do so.

## 1. Introduction

Smoking in pregnancy remains an important public health challenge. In Australia in 2018, 9.2% of women smoked in the first half of pregnancy, the majority (80%) continuing to smoke throughout their pregnancies [1]. The proportions of women smoking in pregnancy are higher in groups already experiencing significant socio-economic disadvantage and marginalisation such as Aboriginal women and teenage mothers [1]. Many pregnant women are motivated to stop smoking but face significant barriers to doing so [2,3,4]. Therefore, assistance with cessation from healthcare providers including midwives and obstetricians is a critical element of healthcare. Guidelines for clinicians to assist women have existed for 15 years in Australia [5] but implementation of these guidelines is sub-optimal [6,7,8,9]. These guidelines are based on the “5As” (Ask, Advise, Assess, Assist and Arrange follow up). Our team developed a comprehensive intervention (the MOHMQuit program—Midwives and Obstetricians Helping Mothers to Quit smoking) using the Behaviour Change Wheel (BCW) to support implementation of these guidelines. Integral to the intervention is a suite of evidence-based materials.

In a paper reporting the development of a resource as part of a nutritional campaign, Everett-Murphy et al. [10] articulate several useful key principles for developing effective materials for use within an intervention. These are: defining the objectives of the intervention and defining the audience; undertaking research to identify the needs and preferences of the audience; using behaviour change theory; and considering the context in which the materials will be used and how the intervention will be implemented.

We set out to develop materials for use by clinicians and maternity service leaders aimed at supporting changes to their behaviour in relation to provision of smoking cessation support and thereby improving the implementation of the Guidelines. Whilst the literature often provides clear description of the development of interventions, including in the context of smoking cessation in pregnancy, using approaches such as the Behaviour Change Wheel (BCW) [11,12,13,14], it contains fewer detailed descriptions of the development of materials (an integral part of many interventions) particularly in contexts where the intervention was aimed at changing clinician behaviour. The aim of this paper is therefore to describe the rationale, process and outcome for developing the MOHMQuit intervention materials.

## 2. Materials and Methods

### 2.1. Process of Developing the Intervention

A detailed description of how the intervention was developed is contained in a separate paper [15]. In brief, the BCW was used to develop the intervention. The BCW has at its centre a behaviour system articulating that the source of behaviour is an interacting combination of capability, motivation and opportunity components. The Wheel then has a middle ring of intervention functions, e.g., training, persuasion, etc. and an outer ring of policy categories [16]. The starting point was to conduct comprehensive mixed methods research across NSW Australia, using the Theoretical Domains Framework (TDF) to identify the significant barriers and enablers for clinicians in implementing the Guidelines [17,18]. The TDF, developed and validated by behavioural scientists, has 14 domains which are distillations of psychological constructs from behaviour change theory [19]. It provides a mechanism to identify contributing barriers and enablers and guide strategies to include in behaviour change interventions. As the TDF maps into the BCW components, its use ensures an evidence-based, structured and comprehensive approach to diagnosing the problem and identifying behaviours that require change. This in turn helps identify the objectives of the intervention and define the audience (clinicians and maternity service leaders)—the first of the Everett-Murphy principles of resource development [10].

Our initial research identified barriers and enablers in five of the six BCW components (psychological capability—having the knowledge and psychological skills to do the task, physical opportunity, social opportunity—social cues and interpersonal influences and the cultural norms of the service, reflective motivation—conscious intentions and evaluations and automatic motivation such as emotional responses, desires and needs), and which behaviours needed change, e.g., not referring women to the Quitline [15]. The research also identified specific barriers relating to materials. These were both physical opportunity barriers in terms of an absence of materials in their service, and psychological capability barriers as clinicians’ expressed preferences for materials that were accurate and based on the current evidence [17]. Clinicians who were unsure about how accurate or current materials for women available in their service were, hesitated to use them. In this way, the best practice principle of identifying the needs and preferences of the audience was adhered to [10]. Through a method outlined in the BCW process, the barriers and enablers were then mapped to intervention functions and then to the behaviour change techniques that were best suited to achieving the clinician behaviour change required, e.g., education and persuasion from a credible source about the effectiveness of Quitlines to facilitate clinicians’ improved referral to the Quitline. Further detail of the mapping processes is contained in our paper on the development of the intervention [15]. Behaviour change theory was therefore integral to the intervention and material development, and use of the TDF to drive the initial research ensured consideration of the context of the target population. These are two key best practice principles [10].

The intervention includes systems changes, e.g., amendments to the electronic database clinicians use for maternity care; leaders and clinicians undertaking pre-training online knowledge-based modules (which cover harms of antenatal smoking; smoking as an addiction; the evidence that smoking cessation support from clinicians is effective and use of NRT in pregnancy); a half-day workshop for leaders and a full day training course for midwives [15]. The intervention is innovative in including elements targeting systems and maternity service leaders as well as clinicians. Consideration of materials was an integral part of the development of the intervention and based on the same analyses as described above.

The research team behind the intervention development consisted of an implementation science specialist (and author of the Behaviour Change Wheel-BCW [16]) Dr Lou Atkins, and four of the five authors on this paper (all with experience of smoking cessation research), i.e., a public health physician (MP), a senior midwife (CA), a social scientist (JL), and a researcher with expertise in behaviour change and implementation science (CP). The fifth author is a smoking cessation expert clinician and researcher (JM). This research team worked closely with a wide range of stakeholders throughout the entire project including in the development of the materials.

### 2.2. The Collaborative Process to Develop the Materials

**Figure 1:**
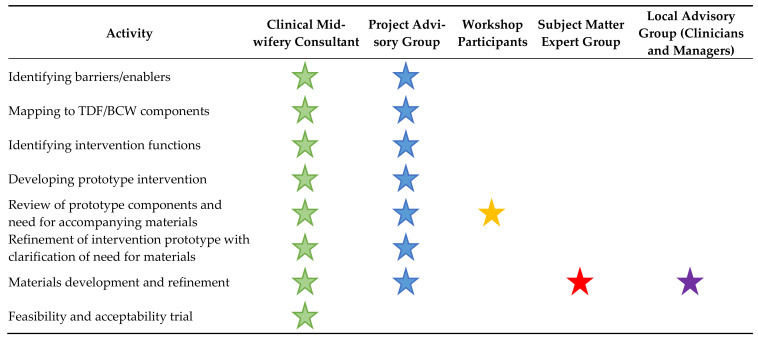
Stakeholder participation in materials development.

Materials were developed in a collaborative process with stakeholders who were maternity leaders at state as well as service level, clinicians, smoking cessation experts, policy makers, academics and pregnant and post-partum women, and their feasibility and acceptability explored in a small local trial. A summary of stakeholder participation is described in Figure 1 below. Early steps such as identifying barriers, mapping to the TDF, to the BCW components and intervention functions and developing the prototype contributed to a developing understanding of which materials might be required and the role they would play in the intervention. There were many ways in which stakeholders were engaged in the work of developing materials as an integral part of the intervention, including as core members of the research team, through a Project Advisory Group, at a large workshop to review potential components of the prototype intervention, a Subject Matter Expert Group and a Local Advisory Group as well as during the feasibility and acceptability trial.

In Australia, each Local Health District has a Clinical Midwifery Consultant, a senior health service position whose role is to provide strategic and operational leadership in maternity services to ensure they are contemporary, evidence informed and responsive to the requirements of the local populations. As described above, the local Clinical Midwifery Consultant was a core member of the research team developing the intervention and therefore the materials.

The research team benefitted from the expertise and experience of an Advisory Group which provided independent advice and direction on the progress and implementation of the project, including the development of the materials, and enhanced relevancy and translation to policy and practice. The Advisory Group consisted of midwives, an obstetrician, maternity leaders at state and local levels, policy makers and academics. The Group met quarterly over a three year period throughout the initial research and the development of the intervention and materials and so had a good understanding of the project and supported a funding application for development of the materials as an integral part of the intervention.

Once the prototype intervention had been developed it was comprehensively reviewed at a day-long face-to-face workshop with 24 attendees consisting of the Advisory Group and additional policy makers, midwives, midwifery managers, educators, cessation specialists and academics. At the workshop, each element of the prototype intervention was scrutinized to assess affordability, practicability, effectiveness, acceptability, side effects, and equity (the BCW APEASE criteria [16]) and ideas for materials needed to accompany each element were discussed. Further detail on the workshop can be found in our related paper [15]. This, as well as feedback from the Advisory Group, ensured close consideration of how the intervention would work practically with clinicians and maternity service leaders (the final best practice principle in developing these types of interventions/materials) [10]. At the workshop it became evident that a series of short video-clips (using actors rather than clinicians and pregnant women) would be needed to support a large number of elements of the intervention. Other needs identified at the workshop included key written materials for clinicians such as prompts/cues to use the 5As, up to date and accurate information on nicotine replacement therapy for clinicians, as well as materials for pregnant women who smoked.

Guided by the BCW process, scripts for video clips were drafted to specifically target the changes in behaviour that were required. Particular choices of words for scripts and the content of materials were based on the preliminary research for developing the MOHMQuit intervention along with existing materials and the extant published literature. The funding for development of the materials covered a part-time project officer; funds for a professional video production company to employ the actors, and film and edit the video clips; and professional graphic design work for all the materials. A Subject Matter Expert Group (the SMEG) comprising the research team, the project manager and two experts in smoking cessation support was convened. The SMEG drafted the scripts for the video clips and developed the materials. The SMEG invested time in identifying and assessing the quality and usability of existing materials from a wide range of international and domestic sources including the state health department. Individual members of the SMEG brought materials to meetings which were reviewed by the SMEG. Quality assessment of these materials by the SMEG was focused on how up to date and accurate the content was and how applicable to an Australian context, along with (particularly in terms of materials for women) how accessible the materials were, for example readability assessed using the Flesch reading ease function in Microsoft Word (using Grade 8 as a guide—plain English and easy to understand by someone aged 13–15). In two instances, existing materials that were excellent and served the exact behaviour change purpose required were integrated into the intervention, but most materials were developed from scratch.

There were many iterations of the scripts (and some minor adjustments were made during filming to improve the realism of the videos) and materials during the refining process including following further consultation with the Project Advisory Group. In addition, a Local Advisory Group of experienced clinicians was convened to review the video clip scripts and the materials, and their feedback was crucial to ensuring accessibility, realism and usefulness. Drafts of the two written materials that were specifically for women, in addition to being reviewed during their development by a wide variety of professional stakeholders, were discussed with a small number of pregnant and postpartum women individually, who smoked and who reviewed the content and visual appeal of the materials. A number of minor adjustments were made to the language used and order in which information was presented in the materials following this review. Finally, a small pilot study exploring the feasibility and acceptability of the intervention with midwifery service leaders and midwives was undertaken. The pilot study took place at one rural hospital. The senior midwifery manager there was supportive of the project and supplied email addresses for leaders and midwives that were used to invite potential participants to take part. For leaders and midwives who provided informed consent to participate, one leaders half-day workshop was run, followed by one full day workshop for midwives. The materials were introduced and discussed at these workshops and then supplies left with the site. The acceptability and feasibility of implementing the leader and midwife components of MOHMQuit, including how much the materials were used and perceptions of their ease of use and usefulness, were assessed using a semi-structured interview with each of the leaders before and three months after the leaders workshop (guided by Sekhon et al.’s framework [20]), and by use of a brief questionnaire with midwives before and after their workshop and again three months later. Midwives were asked to assess the usefulness, and ease of use of the materials on a four point likert scale (from not at all useful to very useful, and from very difficult to very easy to use). The pilot study was granted ethical approval from the Northern NSW Local Health District’s Human Research Ethics Committee (ref: LNR235 2019/ETH10681). Further detail about the small pilot study is included in our paper on the development of the intervention [15].

Ethical approval for the development of the MOHMQuit intervention including the materials was granted from the Hunter New England Human Research Ethics Committee (ref: 14/06/18/5.04).

### 2.3. Outcomes-Materials Developed

Eleven short video clips were developed (ranging from one to 4 min-Table 1), designed as a flexible training resource to be used in the MOHMQuit-specific training, but also at handover and other professional development opportunities. They were planned to promote discussion around supporting pregnant women to stop smoking (they demonstrate/model effective techniques and approaches but are not ‘perfect’), and to be ‘cherry-picked’ rather than watched in any particular order. Each clip had an accompanying written transcript of the dialogue (and non-verbal interaction) within the video. The video clips covered: motivating women to stop smoking; discussing smoking without damaging relationships with women; providing assistance and follow up; stopping not cutting down; integrating smoking conversations into routine clinical care; feedback from women and feedback from midwives.

The materials developed for leaders included two which supported leaders’ assessment of smoking cessation support in their service. Firstly, a data analysis process (a template to run a report from the electronic database used by services to record antenatal care provided), and secondly an audit and action planning tool which addressed topics of level of commitment of staff, motivators and barriers, policies and procedures, teamwork, training, environment and materials for smoking cessation support. Other materials were developed for use in the leaders’ training. These were guidance on developing/maintaining champions; a handout on evidence of effectiveness (of the 5As approach) and a detailed ‘next steps’ handout for the end of training.

Materials for clinicians included several which were aimed at including smoking cessation support in routine antenatal care: a laminated simplified version of the 5As; a reference card aide memoire that clipped to the staff badge (common practice for other topics); a flip top booklet on assisting women and arranging follow up providing suggested phrases and questions for enhancing motivation, setting a date to stop, managing triggers and cravings, and NRT; a double-sided A5 sized ‘helpful hints’ resource covering nicotine addiction and withdrawal, symptoms of withdrawal, reasons women smoke, barriers to stopping and strategies to overcome these; and ‘cheat sheets’ on how to use the electronic database to record smoking cessation support provided. For the clinicians’ training, the video clips; handouts on evidence that women want and expect to be asked about their smoking; the effectiveness of Quitlines; and a handout on motivation were developed.

Two materials were developed for the women themselves: a booklet entitled “Stopping Smoking for You and Your Baby” covering: reasons to stop smoking; good things about stopping smoking; understanding why you smoke; what can help; managing cravings; making a stopping plan; and staying a non-smoker after your baby is born, and a leaflet on Nicotine Replacement Therapy (NRT) covering: why use NRT; types of NRT, how to use them and how much to use.

## 3. Results

### 3.1. Rationale-How Materials Developed Address Identified Need

Table 2 provides a summary of which barriers needed to be addressed and enablers that could be built upon. The first column of each row shows the main barrier or enabler which our foundational mixed research identified [17,18]. As the way these data were collected and analysed was grounded in the TDF, and the TDF maps onto the BCW components, it was straightforward to list the relevant BCW components and TDF domains in the second column for each of the barriers or enablers, e.g., psychological capability and the knowledge domain within the TDF. As described earlier (Section 2.1) the TDF domains and BCW components (third column) were mapped onto the intervention functions, e.g., training, persuading, modelling, enabling, etc., and the specific Behaviour Change Techniques that were most appropriate, e.g., 12.5 adding objects to the environment, 6.1 demonstration of the behaviour (in a video clip in this case), etc. using a process from the BCW. The viability of potential intervention functions was assessed using the APEASE criteria as described in Section 2.2 above. The final column shows which video clips and materials were developed to address these considerations. These included video clips and written materials integral to the training for leaders and clinicians, for leaders to use in reviewing implementation of the guidelines in their service, and for clinicians to use to inform their clinical practice.

As with other implementation studies, e.g., Riordan et al. [21], all materials developed to support the MOHMQuit intervention were badged with the same logo (itself iteratively designed with input from stakeholders), and used the same font and colours as for the State public health system’s electronic database used during antenatal care visits by clinicians, to enhance familiarity and engage users.

### 3.2. Feedback on Materials from the Feasibility Trial

Feedback three months following the feasibility trial indicated that leaders valued the materials, although they had not proactively supported their use by midwives and only one leader had made use of the data analysis process (which had, encouragingly, prompted them to think creatively about mechanisms to make best use of the outputs of the data analysis process). Materials were enthusiastically received by midwives (Table 3) and only minor adjustments were needed to two of the materials in response to their feedback. Whilst midwives’ assessment of the ease of use of materials remained the same over time, in some instances there was a slight decrease in midwives’ mean scores for the materials three months following the workshop compared to immediately following training. With such a small sample (*n* = 11) it is difficult to assess the extent of the decline, and we hypothesized that it was due to the high level of enthusiasm and engagement expressed by midwives immediately following the MOHMQuit workshop which would have waned slightly over time, but did not cause concern about the feasibility or acceptability of the MOHMQuit materials and their usefulness.

## 4. Discussion

This paper provides a detailed description of the process of developing materials integral to a clinician behaviour change intervention (MOHMQuit). The materials were developed using a highly collaborative approach, were based in research evidence and designed to support the intervention which was developed using a theory-driven, comprehensive and careful process. The materials and video clips support this behaviour change intervention in multiple ways, addressing barriers and building on enablers and are mapped to specific behaviour change techniques. The materials were positively received in a small feasibility and acceptability trial of the intervention.

The MOHMQuit video clips and materials were developed in line with the key principles outlined by Everett-Murphy et al. [10]. The background research defined the objectives of the intervention including the audience whose behaviour we aimed to change (clinicians in antenatal care in the NSW public health system) and identified the needs and preferences of the midwives. Using the BCW ensured behaviour change theory underpinned our approach to developing materials and videos targeting very specific behaviour change purposes as described in Table 2. Finally, working collaboratively and iteratively with a wide range of stakeholders ensured robust consideration of the materials and the context in which they would be used and how they would contribute to the implementation of the intervention.

We have learned that whilst time consuming, there are considerable advantages to systematically and collaboratively developing materials which are integral to a behaviour-change intervention. Careful development of materials based in relevant research that is comprehensive, covering off all the domains in the TDF [19] and then mapping to BCW components, intervention functions and behaviour change techniques (as described in Table 2) in addition to asking health professionals what they want and need [22] means reassurance for all those involved in implementing the intervention and using the materials. This reassurance includes that there is an evidence base for the materials themselves, an understanding of the role those materials play and a theoretical basis for how they are effective in supporting the desired behaviour change. Collaborative development, which again is resource intensive, provides reassurance for the research team that the materials developed are both meaningful and context appropriate. Collaboration facilitates the maintenance of engagement of stakeholders in the whole process from initial research to the finished product of the intervention and materials to accompany it, and its implementation. In addition, new learning and consolidation of existing understanding can occur for those involved in developing and refining the materials to ensure they support the desired behaviour change. The value of the collaborative approach is reinforced by those recommending iterative cycles of development where stakeholder input is integral [23,24], and the UK Medical Research Council’s guidance on developing and evaluating complex interventions which describes including appropriate ‘users’ in all stages of the development of the intervention [25].

Flexibility in the use of the materials, for example the short, focused video clips which will ensure their applicability in a range of instances contributes to the flexibility in the implementation of MOHMQuit, adapting to particular circumstances of each different maternity service [15,26]. In addition, the materials contribute to the sustainability of the intervention, for example the leaders’ data analysis process, and audit and action planning tool was designed for regular use to support the monitoring and development of smoking cessation support over time in a service.

It is possible that had the written materials and video clips not been developed in the way described here, in particular the careful targeting to specific barriers, we would have had less confidence in their quality and potential effectiveness as an important dimension of the behaviour change that the MOHMQuit intervention aims to achieve.

## 5. Conclusions

Whilst developing materials in the way described in this paper is resource intensive it offers considerable reassurance and benefits. The next step in the project is a trial of the effectiveness of the intervention in changing clinicians’ behaviour and increasing smoking cessation across eight sites in NSW, which will include seeking specific feedback on the materials as a part of the intervention. The trial begins in late 2021.

## Figures and Tables

**Table 1:** Summary of video clips developed.

Video 1: How to motivate pregnant women who smoke, to stop smoking 3 min 18 s	Scene 1: Vicki (unsure about stopping) 16 weeks 1st visit
Video 1: How to motivate pregnant women who smoke, to stop smoking 3 min 25 s	Scene 2: Gayle (appears not to want to stop) 17 weeks 1st visit
Video 2: How to discuss smoking with women without damaging your relationship with them 2 min 24 s	Scene 1: Sharon (appears not to want to stop) 24 weeks 2nd visit
Video 2: How to discuss smoking with women without damaging your relationship with them 4 min	Scene 2: Sharon (appears not to want to stop, revised approach by midwife) 24 weeks 2nd visit
Video 3: How to provide practical assistance with stopping smoking and how to arrange follow up 3 min 51 s	Scene 1: Jasmine (wants to stop) 16 weeks 1st visit
Video 3: How to provide practical assistance with stopping smoking and how to arrange follow up 3 min 55 s	Scene 2: Jasmine (stopped then relapsed) 24 weeks 2nd visit
Video 3: How to provide practical assistance with stopping smoking and how to arrange follow up 2 min 3 s	Scene 3: Jasmine (has stopped, preventing relapse) 32 weeks 3rd visit
Video 4: Stopping, not just cutting down 2 min 1 s	Scene 1: Vicki (stopping, not just cutting down) 22 weeks 2nd visit
Video 5: How to integrate cessation support into routine clinical activities and save time 1 min	Scene 1: Tiffany (appears not to want to stop) 28 weeks 2nd visit
Video 6: Pregnant women—what it’s like to talk about smoking with my midwives 3 min 26 s	
Video 7: Midwives—how my existing skills help me support women with smoking cessation 4 min 21 s	

**Table 2:** Summary of the barriers, BCW components, intervention functions and behaviour change techniques that the video clips and materials addressed.

Barrier	BCW Component *TDF Domain*	Intervention Function and Behaviour Change Techniques	Video Clips and Print Materials Developed
Clinicians’ knowledge of the Guidelines was poor [17,18]	Psychological capability *Knowledge*	Education 4.1 Instruction on how to perform a behaviour (*information on how and when to provide the 5As*)	Laminated sheet showing simplified 5As as an aide memoire Brief video clips showing midwives using the 5As Evidence of effectiveness of the 5As handout
There was some confusion regarding the value of cutting down versus quitting [17]	Psychological capability *Knowledge*	Education 5.1 Information about health consequences (*provide information on the risks/benefits of quitting vs. cutting down*)	Brief video clip (Video 4) showing midwives discussing the need to stop smoking rather than cut down
Many clinicians reported poor knowledge and communication skills related to Assisting women, in particular: (i) Assisting motivated women with strategies to quit (including use of NRT); (ii) Assisting to motivate women to try to quit who are not currently motivated; (iii) Arranging follow up [17,18]	Psychological capability *Cognitive and interpersonal skills*	Training 4.1 Instruction on how to perform a behaviour (*detailed information on how to assist women with strategies including use of NRT in pregnancy; and how to motivate them*) 6.1 Demonstration of the behaviour (*demonstration of how to perform each of the elements of assisting under varying circumstances*)	Brief video clips showing midwives demonstrating critical techniques in assisting a variety of women and arranging follow up (Video 3) Flip top booklet on assisting women and arranging follow up‘Helpful hints for clinicians’ booklet Reference card to clip to staff badge NRT information for clinicians (existing resource) NRT leaflet for women
There were no mechanisms or systems for clinicians to use to monitor/self-monitor if they were following the 5As [17,18]	Psychological capability *Behavioural regulation*	Education 2.2 Feedback on the behaviour (*provide information on clinic performance providing 5As*) Enablement 1.2 Problem solving/and 1.4 Action planning/and 2.3 Self-monitoring (*encourage midwives to problem solve barriers and solutions to self-monitoring, and make a plan to manage this*)	Feedback to clinicians from leaders’ data analysis process ‘Cheat sheets’ on how to record cessation support provided [The training for midwives includes midwives making a plan for self-monitoring]
Many clinicians thought that the 5As took too long to deliver within the context of a busy antenatal visit [17,18]	Physical opportunity *Environmental context and resources*	Training 4.1 Instruction on how to perform a behaviour (*reiterating 5As designed to be delivered in a short consultation*) 6.1 Demonstration of the behaviour (*how to have 5As discussions whilst doing other clinical duties*)	Brief video clips showing midwives demonstrating critical techniques in providing smoking cessation support in the context of a busy antenatal visit (Video 5)
There were no service-wide systems to identify smokers at subsequent visits and remind clinicians to deliver 5As [17,18]	Physical opportunity *Environmental context and resources*	Environmental restructuring 7.1 Prompts/cues 12.5 Adding objects to the environment (*as above for 7.1; and add posters and colourful, prominent lists in the clinic*)	Laminated sheet showing 5As as an aide memoire Reference card to clip to staff badge ‘Cheat sheets’ on how to record cessation support provided
There was no system to monitor smoking cessation support that women received, for quality assurance purposes [17,18]	Physical opportunity *Environmental context and resources*	Environmental restructuring 12.5 Adding objects to the environment (*develop a reporting system for managers to monitor cessation support provided*) Training 4.1 Instruction on how to perform a behaviour (*train key leaders in use of the reporting system*)	Leaders’ data analysis process Audit and action planning tool
Lack of materials, e.g., printed materials to use with women who smoke were unavailable, out of date or not specific to pregnancy [17,18]	Physical opportunity *Environmental context and resources*	Environmental restructuring 12.5 Adding objects to the environment (*might be printed pamphlets, or links to online resources which can be centrally updated*)	“Stopping Smoking for You and Your Baby” NRT leaflet for women
Lack of leadership for smoking cessation and a lack of champions at all levels including both managers and peers [17,18]	Social opportunity *Social influences*	Enablement 3.1 Social support (unspecified) 12.2 Restructuring the social environment (*leaders encouraging attendance at training; discussion of 5As in meetings*) 2.2 Feedback on behaviour (*develop mechanism to allow leaders to monitor progress on provision of 5As and encourage this at team meetings/display in staff room etc.*)	Audit and action planning tool Guidance on developing/maintaining champions ‘Next steps’ handout for the end of training
Some midwives lacked confidence to deliver the 5As, especially Assisting women who were struggling [17,18]	Reflective motivation *Beliefs about capabilities*	Persuasion 15.3 Focus on past success/ and 15.1 Verbal persuasion about capability (*highlight communication skills midwives have developed in other areas*) B9.1 Credible source (*delivered by senior or other respected midwife*) Incentivisation 10.4 Social reward (*praise for practising behaviour during and between intervention training sessions*) Enablement 3.1 Social support (unspecified)	Brief video clips showing midwives demonstrating critical techniques in assisting a variety of women and arranging follow up (Videos 1–3); brief video clip of women talking about what it’s like to discuss smoking with their midwife (Video 6); brief video clip of midwives reflecting on how their existing skills help them support women (Video 7) Audit and action planning tool Handout on motivation Handout on evidence that women want and expect to be asked about their smoking
Some midwives did not consider referral to Quitline to be effective [18]	Reflective motivation *Beliefs about consequences*	Education 5.1 Information about health consequences (*that referrals to quitline result in x% increase in quit attempts/rates*) Persuasion 9.1 Credible source (*delivered by Quitline staff or other cessation expert*)	Handout on evidence of the effectiveness of Quitline
Some midwives have concerns about damaging the client relationship [17,18]	Reflective motivation *Beliefs about consequences*	Modelling 6.1 Demonstration of the behaviour (*video showing engaged client and effective midwife*) Persuasion 5.1 Information about health consequences/and 5.3 Information about social and environmental consequences/and 6.3 Information about others approval (*professional patient describing health and emotional (not valued) consequences of midwife not addressing their smoking–gives impression OK to keep smoking*) 9.1 Credible source (*above information delivered by a pregnant or postpartum woman who smoked*)	Brief video clip of women talking about what it’s like to discuss smoking with their midwife (Video 6); Brief video clips showing midwives demonstrating critical techniques in assisting a variety of women in a supportive way that reduces risk of damaging the relationship (Video 2)
Framing smoking as a social issue/lifestyle choice rather than an addiction (and therefore not my role) [17]	Reflective motivation *PR&I*	Education 5.1 Information about health consequences (*Impact of nicotine on the brain and role in addiction–pre-training modules*) Persuasion 9.3 Comparative imagining of future outcomes (*comparison with their successful responses to other behavioural issues, e.g., domestic violence*) 13.2 Framing/reframing (*reframing smoking as a behavioural indicator for intervention rather than a ‘lifestyle choice’-pre-training modules*)	Brief video clips showing midwives demonstrating critical techniques in assisting a variety of women in a supportive way that reduces risk of damaging the relationship (Video 2); brief video clip of midwives reflecting on how their existing skills help them support women (Video 7)
Some midwives uncomfortable asking about smoking [17,18]	Automatic motivation *Emotion*	Modelling 6.1 Demonstration of the behaviour (*video showing engaged client and effective midwife*) Persuasion 5.1 Information about health consequences/and 5.4 Information about social and environmental consequences (*professional patient describing health and emotional (not valued) consequences of midwife not addressing their smoking*) 9.1 Credible source (*professional patient*) 5.4 Information about social and environmental consequences (*other midwives feel good about professional role after delivering 5As*) 9.1 Credible source (*midwifery champion*)	Brief video clips showing midwives demonstrating critical techniques in assisting a variety of women; brief video clip of women talking about what it’s like to discuss smoking with their midwife (Video 6); brief video clip of midwives reflecting on how their existing skills help them support women (Video 7)
**Enablers**			
Knowledge of harms associated with smoking was reasonably good [17,18]	Psychological capability *Knowledge*	Education Training Enablement See ‘knowledge’ barriers. No need to address this	
Clinicians have good communication skills generally and are a trusted source of information for women [17]	Psychological capability *Cognitive and interpersonal skills*	Education Training Enablement See ‘confidence’ and ‘skills’ barriers	
5As can be delivered whilst carrying out other clinical tasks [17]	Physical opportunity *Environmental context and resources*	Training See ‘time’ barrier	
The electronic database prompts to ask about smoking at initial visit [17]	Physical opportunity *Environmental context and resources*	Environmental restructuring See ‘systems’ barriers	
Some clinicians reported increased role satisfaction from delivering 5As [17,18]	Reflective motivation *Professional role and identity*	Persuasion See ‘emotion’ barrier	

Table adapted from Table 1 Passey et al. [15].

**Table 3:** Midwives’ mean scores post-training and at 3 month follow up on usefulness and ease of use of materials.

	Post-Training (Out of 4)	Follow-Up (Out of 4)
*Usefulness of each of the materials*		
Video clips	3.5	*
‘Cheat sheets’ on how to record cessation support	3.9	*
Handout on the effectiveness of Quitlines	3.4	*
*Usefulness of the materials in working with women*		
Laminated simplified version of the 5As	4	3.3
Flip top booklet on assisting women and arranging follow up	3.6	3.3
A5 sized ‘helpful hints’ resource	3.7	3.0
Reference card aide memoire that clipped to the staff badge	3.6	3.3
“Stopping Smoking for You and Your Baby”	3.9	3.3
Leaflet on Nicotine Replacement Therapy (NRT) for women	3.8	3.8
*Ease of using MOHMQuit processes and materials in practice*	3.5	3.5

Table adapted from Table 3 Passey et al. [15]. * As these resources were part of the training for midwives they were only asked about this post-training.
